# Vaccination, Variola, Varioloid

**Published:** 1836

**Authors:** 


### II. ANALYTICAL.

---

### Art. I.—Vaccination, Variola, Varioloid.

Any thing that will add to our stock of knowledge in regard to vaccination and varioloid, cannot be otherwise than interesting to the profession at the present. In a former number of the Journal we published the results of repeated vaccinations in 180 persons, seventeen of whom took the disease twice and three thrice. None of these, so far as we are informed, have taken either the small-pox or varioloid, though many of them have often been the inmates of apartments containing patients afflicted with both. It would seem from this experiment, (though it ought to be repeated, for there may have been something peculiar in the matter which we used,) that a single vaccination, although apparently successful, is not sufficient to destroy the susceptibility of the system to variolous affections. It is our present opinion that the seventeen individuals already referred to, would have taken varioloid, had they been exposed to the contagion of either this disease or small-pox, without repeated vaccination. We are supported in this opinion by various facts since communicated to us by Dr. Ridgely, a close observer and successful practitioner of our city. Still it would seem from an extract we are about to make from a notice of a memoir on the nature of varioloid, by Mr. Albert, of the continent of Europe, that the author is opposed to this doctrine. Although he seems to have had ample opportunities of learning the nature of the disease, still he considers it *sui generis, differing entirely from variola.* In relation to the prophylactic powers of vaccination we would just observe that a short time since we were called to visit a lady who had never been vaccinated, and on whom the eruption of small-pox was just making its appearance. When we first saw her she was attended by two children who had been with her an entire day, without having ever been vaccinated. We, however, immediately introduced vaccine matter into their arms in several places, which took the proper effect, and both of the children escaped small-pox. The husband of the lady was attacked about twelve days after his wife, with all the symptoms of small-pox, but no eruption ever appeared. He had been vaccinated in early life. The extract which we make is taken from the *Gazette Medicale.*

W. W.

"The description which Dr. Albert gives, though long, able and minute, is too extended to be transcribed in this place; he considers that the symptoms of variola indicate an affection of the serous membranes; and that the precursory dyspnœa, cephalalgia, vertigo, tinnitus aurium, and erratic pains in varioloid, point out rather an affection of the serous tissues. He asserts that the pustules of varioloid never contain pus, in any period of its existence, nor is it followed by secondary fever; that the pustule of varioloid never gives way, but desiccates; that the dried crust, instead of falling, as in variola, between the third and fifth days, adheres for three weeks; that cicatrices do not follow this exanthema, unless the parts are scratched by the patient. Whilst in variola the chorion becomes the seat of a phlegmonous inflammation, ending in suppuration, followed by loss of substance, varioloid is a lymphatic phlogosis of the most superficial layer of the integuments; hence the conclusion that the two maladies are developed under the influence of different contagious agents.

"Varioloid, according to Dr. Albert, may afflict persons who have had neither variola, nor vaccine. This circumstance, which he considers to be incontestibly proved, staggers the truth of a proposition generally asserted by physicians, *that varioloid is only small-pox modified by vaccination.* For in these cases, says he, in which no modification could have occurred, the examthema, if it had had the least affinity with true variola, would have taken on the variolous, and not the varioloid character.

"Dr. A has seen 19 cases in which varioloid attacked persons who had previously suffered variola. The following remarks are of great value. "True vaccinia preserves against the small-pox, but not against varioloid. Doubts have, it is true, arisen on every side against vaccination, but I believe erroneously, for at the bottom of every case on which such doubts were founded, some error has certainly lurked. Either the vaccine was false, or the disease engendered was varioloid. This, at least, is the result of my experience; in my practice, I have never seen a single individual affected with variola, in whom vaccination had previously been performed with success; but such patients have had the varioloid." The author contends, that this is another proof of dissimilarity between the two exanthemata, and answers the natural objection, that it is precisely the vaccine virus which modifies the variolous virus and produces varioloid, by referring to the cases of those who had had varioloid without previous vaccination. Dr. A. asserts that re-vaccination is not a preservative against varioloid, any more than that first performed, and that in the course of the same epidemic an individual may have both variola and varioloid.

"The distinction made by the author, in proof of his supposition that small-pox and the varioloid differ in kind, that the precursory signs of the one indicate lesion of the mucous tissues, those of the other of the serous, must be considered as overstrained. By his own showing, the latter are common to both forms of disease, and if, indeed, the former be not as marked in varioloid as in variola, it is because the action on the tegumentary surface in it is (in most cases) much less deep seated and severe, and the sympathetic irritation of the gastro-enteric mucous surface much less. The same want of intensity of inflammation of the skin, may account for the absence of pustulation, or, by future observation it must be decided whether, in the worst cases of true varioloid, pus does or does not form in the pustules. Varioloid, by those who embrace the common opinions of the day as to its nature, is admitted to be a much less severe disease than variola; it is no more proof, therefore, of any generic difference than of mere modification, that the prodromi of varioloid are seen to disappear on the first eruption of the pustules, whilst those of variola exist, though with lessened intensity, as Dr. A. asserts. As to the effects on the tissues, the contents of the pustules are at first lymphatic in both disorders, and as we have erysipelatous inflammation in the skin, in one case just reddening it, and in another destroying it, and causing it and the cellular tissue to suppurate, or to slough, both being nevertheless, cases of erysipelas, differing only in degree, so is the varying intensity of variola and varioloid not a proof that they differ otherwise than in degree also.

"It has never been doubted that varioloid, meaning thereby a modified form of small-pox, could occur occasionally in persons neither vaccinated nor variolated. However, it is not common, and the author cites but three instances, one only of which can be considered as conclusive. This, too, is to suppose that no error of diagnosis was in either case committed, but the great accuracy of observation in Dr. Albert, renders this idea improbable. To the medical public we leave the decision of the question, whether the author ought on the strength of these three cases, to assert that varioloid *is not a variola modified by vaccination.*

"The testimony given by Dr. Albert to the value of the process of vaccination, is gratifying. It coincides with that of Dr. Bousquet, whose admirable treatise "De la Vaccine," was fully reviewed in an early number of this Journal, and is in support of the opinions collected by the author of a pamphlet called "A Popular Essay on the Value and Present Condition of Vaccination," published a few months ago in this city, which strongly set forth the propriety of confiding in this prophylactic. It is much to be hoped, that this occurrence of testimony will, ere long, produce a happy effect. Dr. Albert is also of opinion, that vaccination will not preserve against varioloid, though repeated at intervals; but whether, or not, this be true, and that varioloid is a disease of very limited occurrence; or whether, thanks to the generally protective (and always modifying) power of vaccination, few opportunities occur for its developement, we are sure the proportion of those who undergo it, compared with the number of those who are vaccinated, is very small indeed. Dr. A. states a case of a person who had variola and varioloid during the existence of one epidemic, and contends that this case proves his opinion, because no example is as yet known of an individual's being twice visited by variola, in so short a time. Now we are inclined to look upon this as a case of peculiar susceptibility to infection, and think that the circumstance of the varioloid occurring after the variola, is an evidence to the contrary of Dr. Albert's opinion. His last proof he considers irrefragable, because it is assimilated to other known instances in which two different exanthemata, as miliaria and scarlatina, variola and rubeola, etc., have progressed together. It is a case of variola, on the seventh and eighth days of which there appeared pustules on the chest, neck, back, belly, and inferior extremities, which did not contain pus, but turbid lymph, and were the papulæ of varioloid. This is a singular case, and whether we think with the author, or otherwise, it is perhaps impossible to explain the second eruption, developed on the seventh day of the existence of variola. Can a second attack of small-pox occur so early? If so, and the second attack be modified by the former, varioloid would of course appear. But if this be thought improbable, Dr. Albert, on this point, has the best of the analogy, and of the argument."

